# Sodium-Glucose Transporter-2 (SGLT2; SLC5A2) Enhances Cellular Uptake of Aminoglycosides

**DOI:** 10.1371/journal.pone.0108941

**Published:** 2014-09-30

**Authors:** Meiyan Jiang, Qi Wang, Takatoshi Karasawa, Ja-Won Koo, Hongzhe Li, Peter S. Steyger

**Affiliations:** 1 Oregon Hearing Research Center, Oregon Health & Science University, Portland, Oregon, United States of America; 2 Department of Otorhinolaryngology, Seoul National University College of Medicine, Bundang Hospital, Seongnam, Gyeonggi, Republic of Korea; Universtiy of Maryland School of Medicine, United States of America

## Abstract

Aminoglycoside antibiotics, like gentamicin, continue to be clinically essential worldwide to treat life-threatening bacterial infections. Yet, the ototoxic and nephrotoxic side-effects of these drugs remain serious complications. A major site of gentamicin uptake and toxicity resides within kidney proximal tubules that also heavily express electrogenic sodium-glucose transporter-2 (SGLT2; SLC5A2) *in vivo*. We hypothesized that SGLT2 traffics gentamicin, and promotes cellular toxicity. We confirmed *in vitro* expression of SGLT2 in proximal tubule-derived KPT2 cells, and absence in distal tubule-derived KDT3 cells. D-glucose competitively decreased the uptake of 2-(N-(7-nitrobenz-2-oxa-1,3-diazol-4-yl)amino)-2-deoxyglucose (2-NBDG), a fluorescent analog of glucose, and fluorescently-tagged gentamicin (GTTR) by KPT2 cells. Phlorizin, an SGLT2 antagonist, strongly inhibited uptake of 2-NBDG and GTTR by KPT2 cells in a dose- and time-dependent manner. GTTR uptake was elevated in KDT3 cells transfected with SGLT2 (compared to controls); and this enhanced uptake was attenuated by phlorizin. Knock-down of SGLT2 expression by siRNA reduced gentamicin-induced cytotoxicity. *In vivo*, SGLT2 was robustly expressed in kidney proximal tubule cells of heterozygous, but not null, mice. Phlorizin decreased GTTR uptake by kidney proximal tubule cells in *Sglt2^+/−^* mice, but not in *Sglt2^−/−^* mice. However, serum GTTR levels were elevated in *Sglt2^−/−^* mice compared to *Sglt2^+/−^* mice, and in phlorizin-treated *Sglt2^+/−^* mice compared to vehicle-treated *Sglt2^+/−^* mice. Loss of SGLT2 function by antagonism or by gene deletion did not affect gentamicin cochlear loading or auditory function. Phlorizin did not protect wild-type mice from kanamycin-induced ototoxicity. We conclude that SGLT2 can traffic gentamicin and contribute to gentamicin-induced cytotoxicity.

## Introduction

Aminoglycoside antibiotics, like gentamicin, are essential important clinically for treating critical gram-negative bacterial infections, and are frequently used worldwide [1], [2]. Both infants and adults receive gentamicin for bacterial meningitis, endocarditis, septicemia and for prophylaxis in premature births and surgical cases. Unfortunately, the nephrotoxic and ototoxic side-effects of gentamicin therapy remain serious complications, limiting the clinical use of gentamicin [3]. Gentamicin-induced nephrotoxicity, characterized by proximal tubular necrosis without morphological changes in glomerular structures, can cause acute kidney failure and increased morbidity [4], [5]. Acute renal toxicity is largely reversible because kidney tubule cells can proliferate to replace cells lost to aminoglycoside toxicity [6].

The mechanism of gentamicin-induced cytotoxicity is incompletely understood. Gentamicin can induce cell death mechanisms via mitochondrial damage and caspase activation [7]–[9], as well as the generation of toxic levels of reactive oxygen species [10], [11]. Since it is difficult to inhibit the wide variety of cell death mechanisms that may be induced by gentamicin, an alternative strategy to prevent gentamicin-induced cytotoxicity is to block drug entry into cells. Gentamicin and other aminoglycosides are known to enter cells via at least two mechanisms: endocytosis and permeation through non-selective cation channels. In the kidney, the best characterized entry route for lumenal gentamicin is apical endocytosis and trafficking of gentamicin-laden endosomes to the Golgi complex and endoplasmic reticulum (ER) prior to release into the cytosol from the ER [12], [13]. A non-endocytotic entry route for gentamicin into kidney cells has been demonstrated *in vitro* – via permeation of non-selective cation channels, presumptively transient receptor potential (TRP) channels [14], [15]. Proximal tubule cells are presumed to be more pharmacologically sensitive to gentamicin because these cells take up and retain the drug. Distal tubule cells, however, are more resistant to gentamicin, most likely because they do not readily take up or retain gentamicin in the cytoplasm [14], [16]. Another distinguishing feature is the abundant expression of sodium-glucose transporter-2 (SGLT2; a.k.a. SLC5A2) in proximal, but not distal, tubule cells [17], [18].

SGLT2 is a low affinity, high capacity sodium-glucose electrogenic transporter of glycosides expressed in proximal tubules, and is responsible for ∼90% of glucose resorption from the renal ultrafiltrate [18], [19]. Antagonism of SGLT2 activity induces glycosuria [20], [21] and aminoaciduria [22]. Aminoglycosides also induce glycosuria [23], [24], and nephrotoxicity, predominantly within the proximal tubules [25]. The structure of SGLT2 resembles the major facilitator superfamily of transporters with a large, hydrophilic, elastic vestibule, an internal pore diameter of ∼3 nm, and an exit pore (into cytosol) of ∼1.5–2.5 nm [26], [27], sufficiently large to potentially allow permeation by gentamicin. Non-lethal mutations in SGLT2 occur in humans, with little impact on kidney function besides glucosuria and aminoaciduria, with no reported loss of hearing acuity [22], [28], [29]. Several SGLT2 antagonists have been identified, including phlorizin, a hydrolyzable O-glucoside, several non-hydrolyzable antagonists including O-glycosides (sergliflozin [30], remogliflozin [20]) and C-glycosides (dapagliflozin [31], canagliflozin [21], [32]). These non-hydrolyzable antagonists are being, or have been tested, to reverse Type II diabetes in mice [21], [30], [33] and humans [34].

We hypothesized that SGLT2 can traffic gentamicin into cells, and tested whether SGLT2 expression and was required for accelerated onset of gentamicin-induced toxicity in cell lines. If this hypothesis is correct, then loss of the SGLT2 function *in vivo* should reduce cellular uptake of gentamicin and protect against cytotoxicity. If so, this could potentially prevent nephrotoxicity and ototoxicity during gentamicin therapy.

## Materials and Methods

### Ethics Statement

The care and use of all animals reported in this study were approved by the Animal Care and Use Committee of Oregon Health & Science University (IACUC approval #IS00001801).

### Conjugation and purification of GTTR

Gentamicin-Texas Red conjugate (GTTR) was produced as previously described [15], [35]–[37]. Briefly, an excess of gentamicin (Sigma, MO, USA) in 0.1 M potassium carbonate (pH 10) was mixed with Texas Red (TR) succinimidyl esters (Invitrogen, CA) to minimize the possibility of over-labeling individual gentamicin molecules with more than one TR molecule, and to preserve the polycationic nature of the conjugate [38]. After conjugation, reversed phase chromatography, using C-18 columns (Grace Discovery Sciences, IL), was used to purify GTTR from unconjugated gentamicin, and potential contamination by unreacted TR [39]. The purified GTTR conjugate was aliquoted, lyophilized, and stored desiccated, in the dark at −20°C until required.

### Cell culture

The mouse kidney proximal tubule (KPT2) and distal tubule (KDT3) cell lines were generated and characterized as previously described [14], [40]. These cell lines were maintained in DMEM with 10% FBS, without streptomycin or penicillin, at 37°C.

### Competition and inhibition experiments

Cells plated on 8-well chambered coverslips were washed with DMEM twice and incubated as described below. To establish appropriate competition experiments, KPT2 cells were incubated with 0.4 mM 2-(N-(7-nitrobenz-2-oxa-1,3-diazol-4-yl)amino)-2-deoxyglucose (2-NBDG) (Life Technologies, NY, USA) without or with 1∶1, 1∶50 or 1∶1000 molar ratios of D-glucose (Sigma, MO, USA) in DMEM. 2-NBDG (0.4 mM) was also incubated without or with 1∶1, 1∶10 or 1∶50 molar ratios of phlorizin (Pfaltz & Baue, CT, USA) at 37°C for 20 mins. Phlorizin was solubilized in Dimethyl sulfoxide (DMSO) (Final concentration of DMSO in buffer was <0.001%) prior to dilution in buffer to the required concentration. Cells were washed three times with PBS prior to fixation with 4% paraformaldehyde for 15 minutes.

To examine the effect of D-glucose on GTTR uptake by KPT2 cells, cells plated on chambered coverslips were washed with DMEM twice, co-incubated with GTTR (5 µg/mL, gentamicin base, gentamicin: ∼450–477 g/mol; GTTR: ∼1100 g/mol) and 1∶40, 1∶2000 or 1∶40000 molar ratios of D-glucose for 20 minutes in DMEM at 37°C, with 5% CO_2_, then washed with PBS three times to remove GTTR from extracellular media prior to fixation with 4% paraformaldehyde containing 0.5% Triton X-100 (FATX) for 15 minutes at room temperature. To examine the effect of phlorizin on the uptake of GTTR by KPT2 cells, cells were co-incubated with 5 µg/mL GTTR and 1∶5, 1∶10 or 1∶20 molar ratios of phlorizin at 37°C for 20 mins respectively prior to washing and fixation.

To examine the effect of sodium on GTTR uptake by KPT2 cells, Na^+^ free buffer was made up as follows: 140 mM choline chloride, 5 mM KCl, 2.5 mM CaCl_2_, 1 mM MgSO4, 1 mM KH_2_PO4, and 10 mM HEPES (pH 7.4); choline chloride was replaced 140 mM NaCl in Na^+^ buffer. GTTR uptake experiments were performed described as above.

### GTTR uptake and confocal microscopy

The cellular distribution of fluorescence was examined using a Bio-Rad 1024 ES scanning laser system. For each individual set of images to be compared, the same confocal settings were used, with two acquisition images per well, two wells per experimental condition, and each experiment performed at least three times to confirm consistency of experimental data. GTTR fluorescent pixel intensities were obtained by histogram function of the ImageJ software after removal of nuclei and intercellular pixels using Adobe Photoshop. Pixel intensities were statistically compared within each set of images per experiment, and not compared between replicate experiments due to varying acquisition settings to obtain the best dynamic range. To normalize data between experimental sets, the mean intensity was ratioed against the standard (e.g., GTTR only cells) and plotted [15].

### Immunofluorescence

For immunolocalization of SGLT2, paraformaldehyde-fixed cells were washed in PBS, immunoblocked in 1% serum in PBS for 30 min and incubated with polyclonal anti-SGLT2 antisera (rabbit, Abcam, MA, USA; or goat, Santa Cruz Biotechnology, TX, USA) at room temperature for 1 hour. After washing with 1% serum in PBS, specimens were further incubated with 1∶200 Alexa-488-conjugated goat-anti-rabbit or donkey anti-goat antisera (Invitrogen, CA) for 1 hour at room temperature, washed, post-fixed with 4% paraformaldehyde for 15 min, rinsed and mounted under coverslips with VectorShield (Vector Labs, CA). In vitro studies, when double-labeled for SGLT2 plus GTTR, cells were permeabilized by 0.5% Triton X-100 after immunolabeling.

### Immunoblotting

Kidney and cochlear tissues were analyzed by immunoblot as described before [41]–[44]. Briefly, total protein extracts were prepared by homogenizing tissues in T-PER tissue protein extraction buffer (Thermo Scientific, IL, USA) with protease inhibitor (Sigma, MO, USA), and the total protein concentration determined using the bicinchoninic acid (BCA) assay. Protein samples (100 µg) were separated by 4–20% pre-cast polyacrylamide gel (Bio-Rad, CA, USA), transferred to polyvinylidene difluoride membranes (Millipore Corporation, MA, USA), blocked with 5% non-fat milk and then incubated at 4 C overnight with goat (1∶50; Santa Cruz, CA, USA) or rabbit (1∶50; Abcam, MA, USA) polyclonal antibodies against SGLT2 in 5% non-fat milk. Rabbit polyclonal antibodies against anti-actin (1∶1000; Sigma, MO, USA) were also used as an internal standard. Peroxidase-conjugated anti-goat (1∶1000) or anti-rabbit (1∶2500) antisera were used to localize primary antisera and visualized using an ECL-Plus detection kit (Thermo Scientific, IL, USA), documented with a photoscanner and analyzed with the Fiji (freeware) program.

### KDT3-SGLT2 cell line generation

Mouse SGLT2 cDNA from Open Biosystems (Clone ID: 4235707) was amplified by PCR, using primers *5′-TTT GAA TTC GCC ACC ATG GAG CAA CAC GTA GAG-3′* and *5′-CCC GTC GAC TTA TGC ATA GAA GCC CCA GAG-3′*, digested with EcoRI/SalI, and subcloned into pBabe-puro vector. The resultant plasmid was transfected into Phoenix Eco packaging cell using Lipofectamine 2000. After 48 hours, the retrovirus-containing medium was collected, diluted (1∶500) with growth medium and added to mouse kidney distal tubule KDT3 cells in DMEM with 10% FBS. Culture medium was changed again after 24 h and puromycin was added at 2.5 µg/ml to select for retrovirus-infected cells. From dozens of surviving cells after several days of puromycin treatment, several clones were selected, expanded and used for GTTR uptake experiments as described above. Puromycin was not applied during GTTR uptake experiments.

### Transfection and cell viability measurement

Cell viability was determined by the reduction of 3-(4,5-dimethylthiazol-2-yl)-2,5-diphenyltetrazolium bromide (MTT), an indicator of mitochondrial dehydrogenase activity, as previously described [40], [45]. Briefly, KPT2 cells were plated at 3000 cells per well in a 96-well plate. After incubation overnight to allow cells to attach to the plate, cells were treated with small interfering RNA (siRNA) and control for SGLT2 (Invitrogen, CA). Transfection of siRNA was performed using Lipofectamine RNAiMAX (Invitrogen, CA). After 48 hours, transfected cells were treated with gentamicin (5 or 10 mM) in DMEM (10% FBS) for 1, 2 or 3 days. Subsequently, 20 µl of 5 mg/ml MTT solution was added to each well, and cells incubated for 4 h at 37°C, 5% CO_2_. Culture medium was then replaced with 200 µl DMSO in each well and the optical density recorded at 540 nm with background subtraction at 660 nm. Student's t-test was used for statistical analysis [40], [45].

### Mice

*Sglt2^+/−^* mice were obtained from the Wellcome Trust Sanger Institute (Hinxton, Cambridge, UK), and an in-house colony established from these founders. Homozygous mice were generated either by crossing heterozygotes together or by crossing heterozygotes with homozygotes. Littermates of wild-type and heterozygotes served as controls. A PCR-based genotyping method was used to identify mutant and wild-type alleles. The mutant allele was identified using primers Slc5a2_55706_F: *5′- AGC AGG AGG GTT CAG GCA GG -3′* and CAS_R1_term_x: *5′-TCG TGG TAT CGT TAT GCG CC -3′* (172-bp product). The wild type allele was identified using primers Slc5a2_55706_F and Slc5a2_55706_R: *5′-TTT TGC GCG TAC AGA CCA TC -3′* (412-bp product).

Mice (21–28 days old) received an intra-peritoneal (i.p.) injection of 800 mg/kg phlorizin (200 µg/µl phlorizin in 40% DMSO, pH 7.4; 4 µl/g), or the vehicle alone. Thirty minutes later, mice received an i.p. injection of 2 mg/kg GTTR (in sterile PBS, pH 7.4). After a further 30 minutes, cardiac serum was collected from deeply-anesthetized mice prior to cardiac perfusion with PBS, then 4% paraformaldehyde. Kidneys and cochleae were excised and post-fixed in FATX, and processed for immunofluorescence [39].

### Determination of gentamicin levels in serum and in cells

Serum levels of gentamicin and the gentamicin epitope of GTTR were determined via enzyme-linked immunosorbent assay (ELISA). Serum supernatant was further diluted, centrifuged and protein extracted as needed for ELISA. Measurement of total gentamicin levels in serum was determined according to the manufacturers' instructions (EuroProxima, Arnhem, the Netherlands).

For cellular levels of gentamicin or the gentamicin epitope of GTTR, KPT2 cells were plated in 60 mm dishes and incubated at 37°C, with 5% CO_2_ overnight. After washing with DPBS, cells were incubated in 5 µg/mL GTTR or 1 mM gentamicin and 1∶5, 1∶10 or 1∶20 molar ratios of phlorizin respectively, as described above. After 20 minutes, cells were washed with DPBS three times and proteins extracted. The quantity of cell protein was measured by BCA protein assay kit. Gentamicin ELISAs were performed described as above.

### Auditory testing

ABR thresholds to pure tones were obtained to evaluate hearing function. Wild-type, *Sglt2^+/−^* and *Sglt2^−/−^* mice were anesthetized and placed on a heating pad in a sound-proof, electrically isolated chamber. Needle electrodes were placed subcutaneously below the test ear, at the vertex, and with a ground on the claw. Each ear was stimulated separately with a closed tube sound delivery system sealed into the ear canal. The auditory brain-stem response to a 1-ms rise-time tone burst at 4, 8, 16, 24, and 32 kHz was recorded. Threshold was defined as an evoked response of 0.2 mV [46], [47]. ABR thresholds were obtained both before and 30 minutes after phlorizin treatment in wild-type mice, and in *Sglt2^+/−^* and *Sglt2^−/−^* mice 6 and 12 weeks of age. In addition, ABRs were also obtained before and after aminoglycoside treatment.

### Toxicity studies

Since immunofluorescence may not detect SGLT2 in the cochlea, toxicity studies with aminoglycosides in the presence or absence of phlorizin were conducted. Dosing with gentamicin to induce ototoxicity *in vivo* causes systemic toxicity in mice, therefore wild-type mice were treated with a similar aminoglycoside - kanamycin in the presence or absence of phlorizin [48]. ABR thresholds were obtained before kanamycin dosing. Four groups of mice were used: group 1, sterile Dulbecco's PBS (DPBS) only in the same delivery routes as for subsequent groups; group 2, 800 mg/kg kanamycin in DPBS twice daily, subcutaneously, for 14 days; group 3, 800 mg/kg kanamycin in DPBS twice daily, subcutaneously, plus DMSO vehicle only, i.p., for 14 days; group 4, 800 mg/kg kanamycin in DPBS twice daily, subcutaneously, plus phlorizin (100 mg/kg in DMSO, i.p.) for 14 days. Phlorizin was injected twice daily 15 minutes prior to each kanamycin injection. Subsequently, mice were allowed to recover for 3 weeks before final ABR thresholds were obtained to determine any permanent ABR threshold shift and mice euthanized.

### Statistics

All *in vitro* experiments were performed multiple times to validate the observations, with the data expressed as means ± SEM. Statistical analysis was conducted using the nonparametric *t* test for comparison of 2 groups or ANOVA for comparisons of 3 groups (GraphPad Prism). For *in vivo* experiments, cytoplasmic GTTR fluorescence in kidney proximal tubules was compared between phlorizin treatment and control group. ABR thresholds (or threshold shifts) at each tested frequency were compared between *Sglt2^−/−^* mice and control mice, or between treatment groups in the kanamycin toxicity study, by nonparametric t-test (GraphPad Prism). A confidence level of 95% was considered statistically significant. *p<0.05 and **p<0.01.

## Results

### Uptake of a fluorescent glucose analog, 2-NBDG, was inhibited by phlorizin, an SGLT2 antagonist

**Figure 1:**
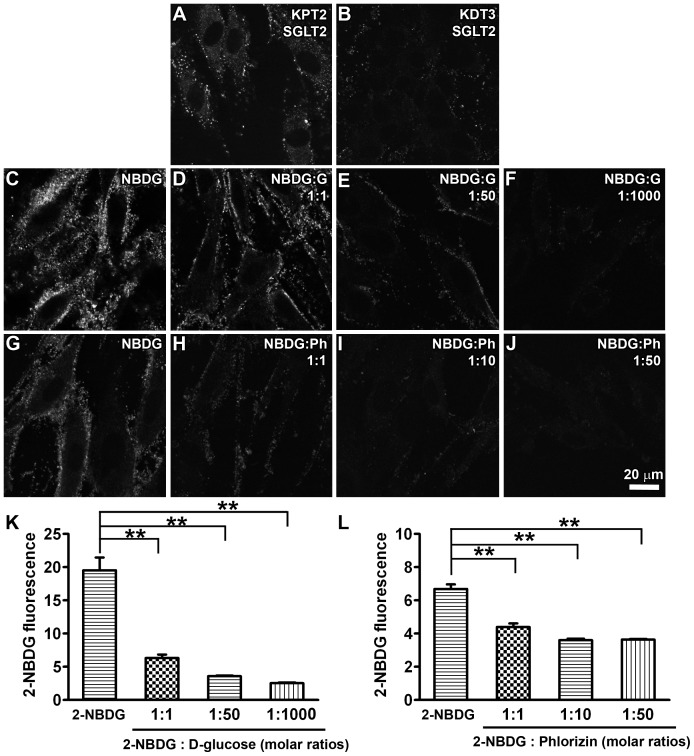
Uptake of the fluorescent glucose analog 2-NBDG is mediated by SGLT2 in KPT2 cells. KPT2 cells (A) had robust SGLT2 immunolabeling compared to KDT3 cells (B). Increasing doses of (C–F) D-glucose (molar ratios of 1∶0, 1∶1, 1∶50 or 1∶1000 [2-NBDG/D-glucose]), or (G–J) phlorizin (molar ratios of 1∶0, 1∶1, 1∶10 or 1∶50 [2-NBDG/phlorizin]) dose-dependently decreased 2-NBDG fluorescence in KPT2. Scale bar = 20 µm. (K, L). The fluorescence intensity of 2-NBDG in KPT2 cells was significantly decreased with increasing doses of D-glucose (K) or phlorizin (L; **p<0.01).

We verified the presence (or absence) of SGLT2 immunoexpression in previously-characterized murine KPT2 and KDT3 cell [14]. SGLT2 was specifically immunolocalized at the periphery of KPT2 cells, but not KDT3 cells (Fig. 1A, B, respectively), presumptively at the cell membrane. Cellular uptake of the fluorescent glucose analog, 2-NBDG, is mediated by both SGLTs and also by facilitated glucose transporters (GLUTs) [49]. In KPT2 cells, 2-NBDG fluorescence was primarily localized at the cell periphery (Fig. 1C). Increasing concentrations of D-glucose (Fig. 1 C–F, K), or the SGLT2 antagonist phlorizin (Fig. 1 G–J, L), dose-dependently reduced 2-NBDG fluorescence in KPT2 cells. This demonstrated the presence of robust SGLT2 activity in KPT2 cells.

### SGLT2-mediated uptake of GTTR by KPT2 cells can be competitively inhibited

**Figure 2:**
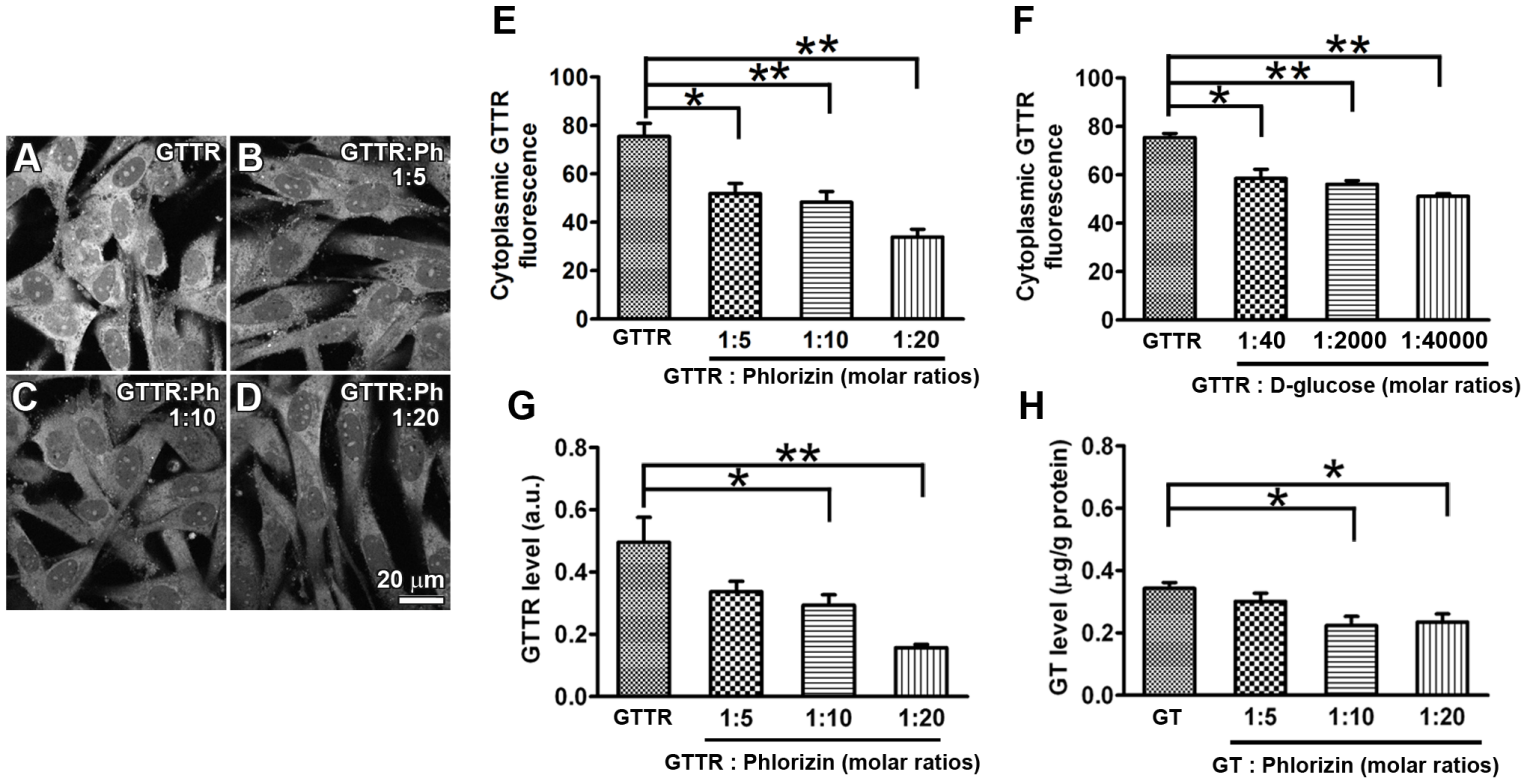
SGLT2-mediated uptake of GTTR can be competitively inhibited. (A–D) Cells were treated with 5 µg/ml GTTR for 20 minutes at 37°C with a dose-range of phlorizin (molar ratios of 1∶0, 1∶5, 1∶10 or 1∶20 [GTTR∶phlorizin]) in DMEM buffer. Scale bar = 20 µm. Increasing doses of (E) phlorizin or (F) D-glucose (molar ratios of 1∶0, 1∶40, 1∶2000 or 1∶40000 [GTTR∶D-glucose]) reduced GTTR fluorescence in KPT2 cells (*p<0.05; **p<0.01). Cell ELISAs demonstrated that (G) GTTR or (H) gentamicin levels in KPT2 cells are decreased by increasing doses of phlorizin.

GTTR is a fluorescently-tagged gentamicin conjugate used to visually test for gentamicin permeation of non-selective cation channels into cells [15], [50]–[53]. We used phlorizin, an SGLT2 antagonist [54] or D-glucose to test whether SGLT2 was potentially GTTR-permeation. Increasing doses of phlorizin (Fig. 2 A–E) and D-glucose (Fig. 2 F, Fig. S1) significantly decreased GTTR fluorescence in KPT2 cells. We then used ELISA technology to verify the imaging data, and found that phlorizin reduced both GTTR and native gentamicin uptake by KPT2 cells in a dose-dependent manner (Fig. 2 G, H), validating GTTR as a tracer for gentamicin studies. Thus, SGLT2-mediated uptake of GTTR by KPT2 cell can be antagonized or competitively-inhibited.

### SGLT2-mediated uptake of GTTR by KPT2 cells can be inhibited by Na^+^ free buffer

**Figure 3:**
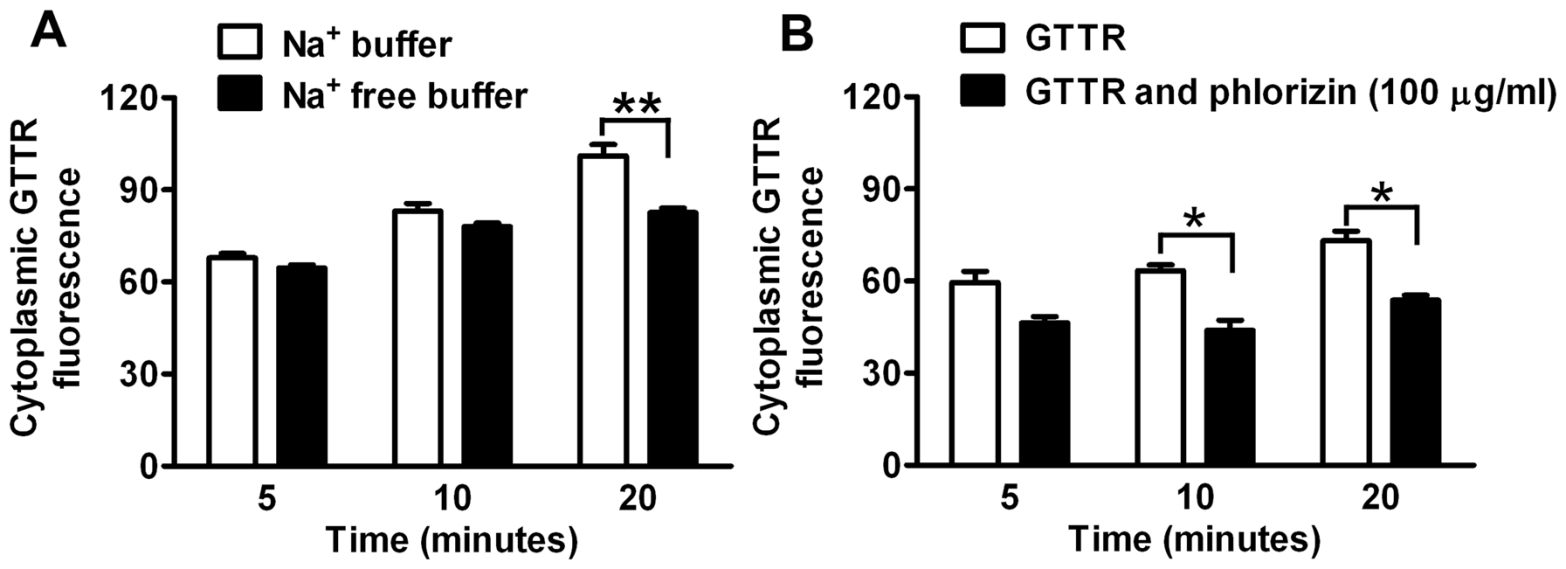
SGLT2-mediated uptake of GTTR by KPT2 cells was inhibited by Na^+^ free buffer. (A) KPT2 cells were incubated with GTTR for 5 minutes, 10 minutes or 20 minutes at 37°C in Na^+^ free buffer or Na^+^ buffer. GTTR fluorescence of KPT2 cell in Na^+^ buffer for 20 minutes was more intense than in Na^+^ free buffer (**p<0.01). (B) KPT2 cells were treated with GTTR and phlorizin in DMEM buffer. GTTR uptake by KPT2 cells was also inhibited by phlorizin (100 µg/ml) over time (*p<0.05).

SGLT2 is a Na^+^-ligand symporter [55]. We examined whether GTTR uptake by KPT2 cells was attenuated in Na^+^-free buffer after 5, 10 or 20 minutes at 37°C. GTTR fluorescence in KPT2 cells in Na^+^ -free buffer was significantly attenuated (∼20%) after 20 minutes (Fig. 3 A). We used phlorizin to further verify this data over time. Phlorizin also significantly inhibited ∼20% GTTR uptake of KPT2 cells, most consistently at the 20 minute timepoint (Fig. 3 B), and this timepoint was chosen for the majority of subsequent experiments. Thus, SGLT2 accounts for ∼20% of total GTTR uptake in SGLT2-expressing KPT2 cells.

### Enhanced GTTR uptake by KDT3 cells heterologously expressing SGLT2

**Figure 4:**
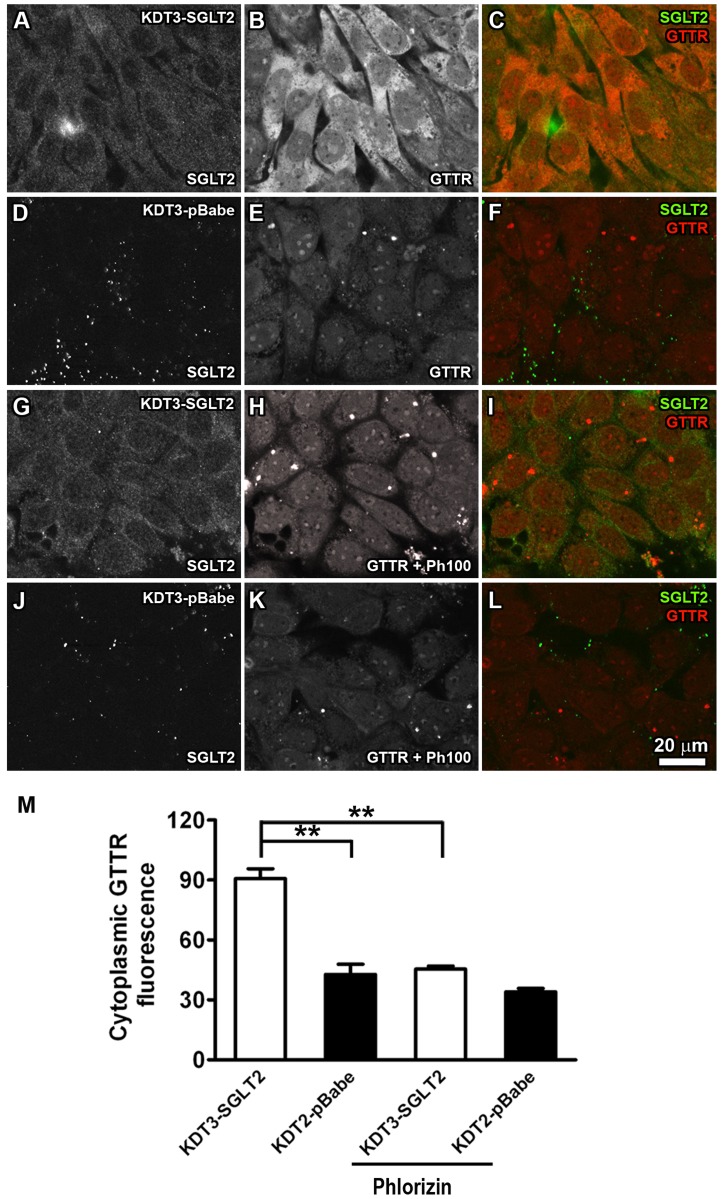
Heterologous expression of SGLT2 in KDT3 cells increased cellular uptake of GTTR. (A–C) KDT3-SGLT2 cells with positive SGLT2 immunofluorescence displayed robust GTTR uptake (B, C). (D–F) Empty vector control clones (KPT2-pBabe) showed negligible SGLT2 immunofluorescence (D) and weak, uniform levels of GTTR fluorescence (E, F) compared to (B, C). (H, I) GTTR fluorescence in KDT3-SGLT2 cells in the presence of phlorizin (100 µg/ml) was visibly less intense than in KDT3-SGLT2 cells without phlorizin treatment (B, C). (K, L) GTTR fluorescence in phlorizin-treated KDT3-pBabe cells showed weak levels of GTTR fluorescence as untreated in KDT3-pBabe cells (E, F). Scale bar = 20 µm. (M) Fluorescence intensities of GTTR in KDT3-SGLT2 or KDT3-pBabe cells in the presence or absence of phlorizin (100 µg/ml; **p<0.01).

To test if SGLT2 can enhance cellular uptake of GTTR, stable cell lines expressing SGLT2 were generated using KDT3 cells that do not endogenously express SGLT2 (Fig. 1 B). KDT3-derived cell lines expressing SGLT2 (KDT3-SGLT2) and empty vector control cell lines (KDT3-pBabe) retained the parental KDT3 morphology (Fig. S2). Immunofluorescence revealed expression of SGLT2 in most KDT3-SGLT2 cells, with negligible immunofluorescence for SGLT2 in KDT3-pBabe cells (Fig. 4 A, G and D, J respectively).

Following a 20 minute incubation with GTTR, robust GTTR uptake was present in KDT3-SGLT2 cells immunolabeled for SGLT2, but not in control KDT3-pBabe cells lacking SGLT2 immunofluorescence (Fig. 4 B, E). Pixel intensity analysis revealed statistically significant increases in GTTR fluorescence within KDT3-SGLT2 cells compared to that of control KDT3-pBabe cells (Fig. 4 M). In the presence of phlorizin (100 µg/ml), GTTR fluorescence in KDT3-SGLT2 cells was significantly less than in KDT3-SGLT2 cells treated without phlorizin (Fig. 4 B, H, respectively). In addition, GTTR fluorescence in phlorizin-treated KDT3-SGLT2 cells was not significantly different to KDT3-pBabe cells with or without phlorizin treatment (Fig. 4 M), demonstrating the specificity of phlorizin for SGLT2 in these cells. Thus, exogenous expression of SGLT2 in KDT3 cells facilitated GTTR uptake.

### Knock-down of SGLT2 reduced gentamicin-induced cytotoxicity

**Figure 5:**
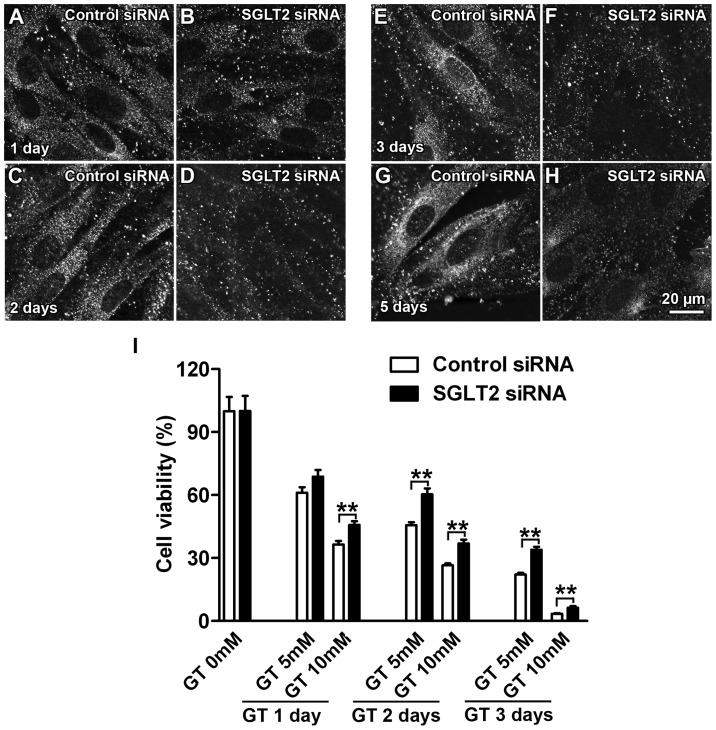
Knockdown of SGLT2 reduced gentamicin-induced cytotoxicity. (A–H) KPT2 cells transfected with siRNA for SGLT2 showed reduced immunoexpression of SGLT2 compared with cells transfected with control siRNA. (A–D) The effect of SGLT2 siRNA began within 1 day of transfection and was most apparent 2 days of transfection. (A–H) The effect SGLT2 siRNA tranfection lasted for at least 5 days. (I) MTT assay on cells (2-days post-transfection) treated with gentamicin for 1, 2 or 3 days revealed greater viability of SGLT2 siRNA-transfected KPT2 cells compared with KPT2 cells treated with control siRNA (**p<0.01).

To test whether SGLT2 contributes to gentamicin-induced cytotoxicity, we transfected KPT2 cells with siRNA for SGLT2 to knock-down protein expression of SGLT2 prior to drug exposure. Immunofluorescence confirmed that SGLT2 siRNA reduced SGLT2 expression compared to control siRNA-transfected cells (Fig. 5 A–H). The effect of SGLT2 siRNA was apparent 1 day after transfection and further reduced SGLT2 expression 2 days after transfection (Fig. 5 A–D). This knock-down of SGLT2 expression lasted at least 5 days (Fig. 5 A–H). Two days after transfection with control or SGLT2 siRNA, KPT2 cells were treated with gentamicin (5 mM or 10 mM) for 1, 2 or 3 days, prior to MTT assay for cell viability [40], [45]. Control and SGLT2 siRNA-transfected cells showed no difference in viability (Fig. S3), demonstrating that loss of SGLT2 did not affect cell viability. Although gentamicin reduced cell viability, SGLT2 knock-down attenuated the degree of gentamicin-induced toxicity, most significantly at 2 or 3 days of gentamicin treatment (Fig. 5 I). Thus, KPT2 cells with SGLT2 expression were more susceptible to gentamicin-induced cytotoxicity than KPT2 cells with SGLT2 knock-down, suggesting that SGLT2 trafficking of gentamicin contributed to gentamicin-induced cytotoxicity.

### Immunoexpression of SGLT2 in renal and cochlear tissues *in vivo*

**Figure 6:**
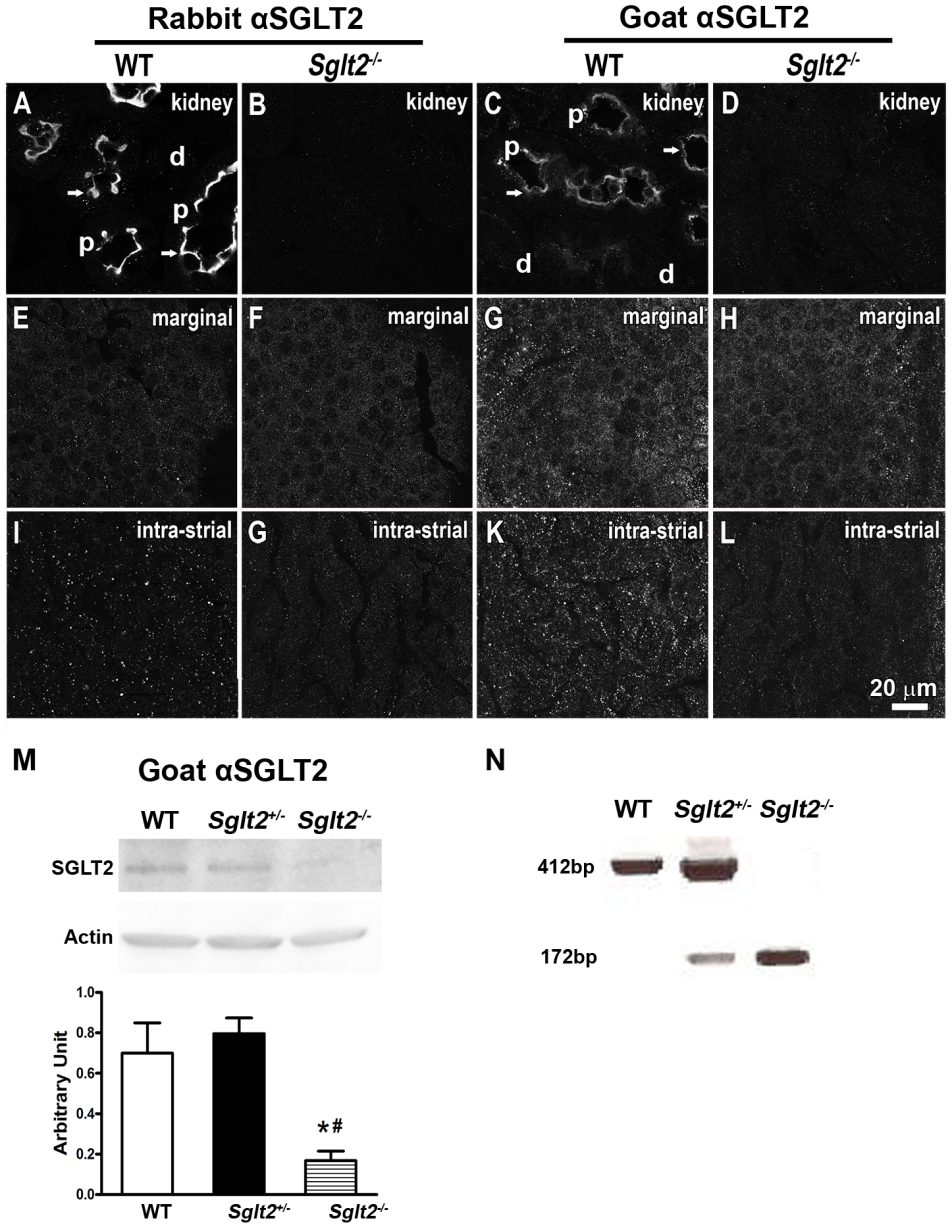
SGLT2 immunofluorescence in the kidney and cochlea. Two different SGLT2 antibodies were used, a rabbit polyclonal IgG to synthetic peptide derived from residues 250–350 of human SGLT2 and a goat polyclonal IgG against a murine peptide sequence within the N-terminal extracellular domain of SGLT2. (A, C) In wild-type mice, SGLT2 was immunolocalized at the apical membranes (arrows) of proximal tubules (p), but not in adjacent glomerular (not shown) or distal tubule (d) regions. (B, D) In *Sglt2^−/−^* mice, no immunoexpression for renal SGLT2 was observed with either antibody. (E, F) No labeling above background was observed in cochlear marginal cells of wild-type or *Sglt2^−/−^* mice with rabbit antisera for SGLT2. (G, H) Goat antisera for SGLT2 produced labeling patterns in cochlear marginal cells of both wild-type mice and *Sglt2^−/−^* mice, suggestive if substantial non-specificity in this cell type. (I, K) In the intra-strial layer of wild-type mice, predominantly composed of both marginal and intermediate cells, both antisera exhibited a punctate labeling pattern not observed in *Sglt2^−/−^* mice (J, L). Scale bar = 20 µm. (M) Immunoblotting with the goat antibody for SGLT2 revealed SGLT2 protein expression in wild-type and *Sglt2^+/−^* mice, but not *Sglt2^−/−^* mice. The ratio of SGLT2 to actin expression in kidney tissues of wild-type and *Sglt2^+/−^* mice were significantly higher than that in *Sglt2^−/−^* mice. There was no statistical difference in SGLT2 protein expression between wild-type and *Sglt2^+/−^* mice. (N) Genotyping demonstrated the absence of wild-type SGLT2 alleles in *Sglt2^−/−^* mice.

To determine if SGLT2 is appropriately located for gentamicin uptake *in vivo*, the immunoexpression of SGLT2 was characterized in fixed renal proximal tubules and cochleae *in situ* using two different antibodies. In the kidney, as previously described [17], [18], [56], SGLT2 was immunolocalized at the apical brush border membranes of wild-type renal proximal tubule cells, but not in adjacent distal tubule regions (Fig. 6 A, C), nor in the kidney of *Sglt2^−/−^* mice (Fig. 6 B, D). In the cochlea, the rabbit anti-SGLT2 antibody did not label wild-type marginal cells above background (Fig. 6 E), or exhibited non-specificity in marginal cells of *Sglt2^−/−^* mice (Fig. 6 F). The goat anti-SGLT2 antibody consistently exhibited non-specific fluorescence in marginal cells of *Sglt2^−/−^* mice (Fig. 6 H) similar to that observed in wild-type marginal cells (Fig. 6 G). Both SGLT2 antibodies consistently exhibited a punctate labeling pattern within the intra-stria vascularis (Fig. 6 I, K) that was not present in *Sglt2^−/−^* mice (Fig. 6 G, L). Immunoblotting revealed SGLT2 protein expression in kidneys of wild-type and *Sglt2^+/−^* mice, but not in *Sglt2^−/−^* mice (Fig. 6 M). Immunoblotting of wild-type cochlear tissues detected actin, but not SGLT2 (data not shown), indicative of the low level expression of SGLT2 protein in cochlear tissues. PCR-based genotyping demonstrated the absence of wild-type alleles in *Sglt2^−/−^* mice (Fig. 6 N).

### Phlorizin decreased renal uptake and increased serum levels of GTTR *in vivo*

**Figure 7:**
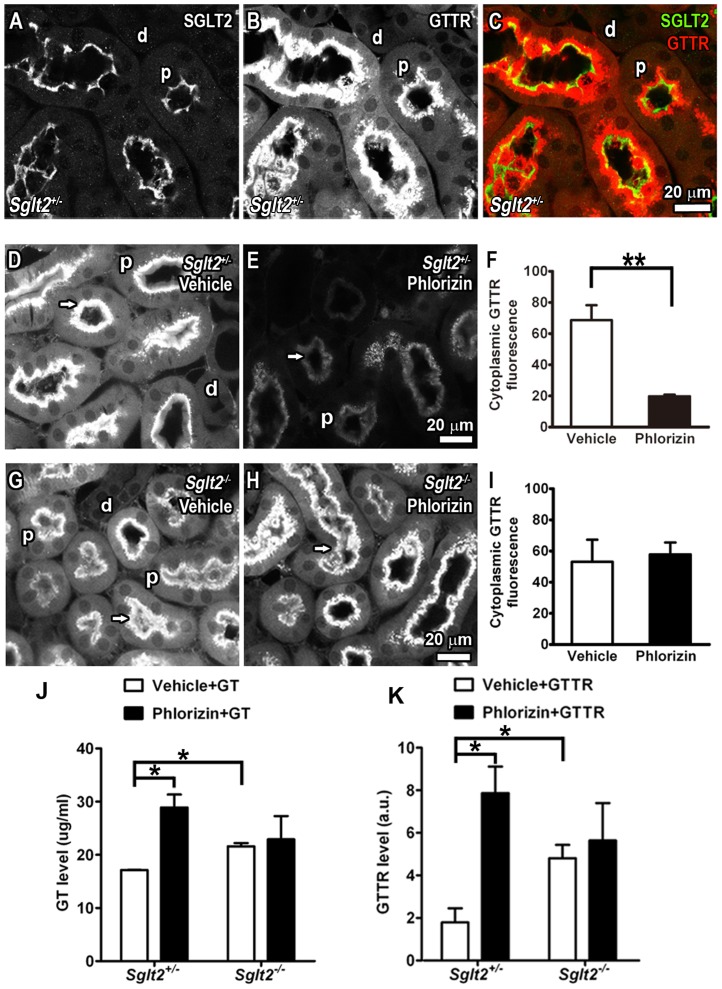
Phlorizin decreased renal GTTR uptake, and increased serum drug levels *in vivo*. (A) In *Sglt2^+/−^* mice, rabbit anti-SGLT2 immunolabeling was predominantly localized at the apical, lumenal region of proximal tubules (p), with negligible labeling in distal tubules (d). (B) GTTR fluorescence was most intense (as saturated puncta) in the apical region of proximal tubules (p), with less intense diffuse labeling in the cytoplasm of these same cells. Very weak and only diffuse GTTR fluorescence was observed in the cytoplasm of distal tubule cells (d). (C) Merged image showing colocalization of SGLT2 (green) and GTTR (red) in proximal tubules. (D–F) When *Sglt2^+/−^* mice were pre-treated with phlorizin, significantly reduced GTTR fluorescence was observed in the cytoplasm and apical brush border (arrows) of proximal tubule cells (E) compared to untreated mice (D, F; **p<0.01). (G, I) In *Sglt2^−/−^* mice, GTTR fluorescence was diffusely distributed throughout the cytoplasm of proximal tubule cells, with intense fluorescence at the apical brush border. (H, I) Phlorizin had no effect on the uptake, distribution or intensity of GTTR fluorescence in *Sglt2^−/−^* proximal tubule cells (**p<0.01). Scale bar = 20 µm. (J, K) In *Sglt2^+/−^* mice, phlorizin pre-treatment significantly increased both gentamicin and GTTR serum levels compared to vehicle treated control mice (*p<0.05). In *Sglt2^−/−^* mice, phlorizin did not significantly change gentamicin or GTTR serum levels. However, serum levels of gentamicin or GTTR serum level were significantly higher in *Sglt2^−/−^* mice than in *Sglt2^+/−^* mice in the absence of phlorizin treatment (*p<0.05).

Since phlorizin had no effect on the bactericidal activity of gentamicin on *E. coli* by disk diffusion assay (Table S1), the *in vitro* data suggested that phlorizin may decrease cellular uptake of GTTR *in vivo* and potentially reduce aminoglycoside-induced cytotoxicity *in vivo*. To test whether phlorizin decreased cellular uptake of GTTR *in vivo*, the intensity of cytoplasmic GTTR fluorescence was determined in proximal tubule cells of *Sglt2^+/−^* and *Sglt2^−/−^* mice. In *Sglt2^+/−^* mice, rabbit anti-SGLT2 immunolabeling was co-localized with GTTR fluorescence in proximal, but not distal tubule cells (Fig. 7 A–C). GTTR fluorescence was diffusely distributed throughout the cytoplasm, and intensely localized at the brush border of proximal tubule cells of *Sglt2^+/−^* mice that received GTTR plus vehicle only *in vivo* (Fig. 7 D). Phlorizin pre-treatment visibly reduced cytoplasmic GTTR fluorescence within proximal tubule cells and at their brush border (Fig. 7 E, F). In *Sglt2^−/−^* mice, unexpectedly, GTTR fluorescence was diffusely distributed throughout the cytoplasm, and intense fluorescence at the brush border of proximal tubule cells (Fig. 7 G), as observed in untreated *Sglt2^+/−^* mice (Fig. 7 D). Phlorizin had no significant effect on the intensity (uptake) or distribution of GTTR fluorescence in proximal tubule cells of *Sglt2^−/−^* mice (Fig. 7 H, I). Thus, SGLT2 is not required for renal proximal tubule uptake of GTTR in *Sglt2^−/−^* mice, although GTTR uptake by these cells can be acutely inhibited by the SGLT2 antagonist, phlorizin, in *Sglt2^+/−^* mice.

In *Sglt2^+/−^* mice, phlorizin increased serum levels of both gentamicin and GTTR levels compared to vehicle-treated mice (Fig. 7 J, K). In *Sglt2^−/−^* mice, phlorizin had no effect on serum levels of gentamicin or GTTR (Fig. 7 J, K). Gentamicin and GTTR serum levels in *Sglt2^−/−^* mice were significantly higher than in *Sglt2^+/−^* mice (Fig. 7 J, K). Thus, loss of SGLT2 function, by antagonism, or by gene deletion, increases serum levels of gentamicin.

### Phlorizin did not affect cochlear uptake of GTTR or auditory function

**Figure 8:**
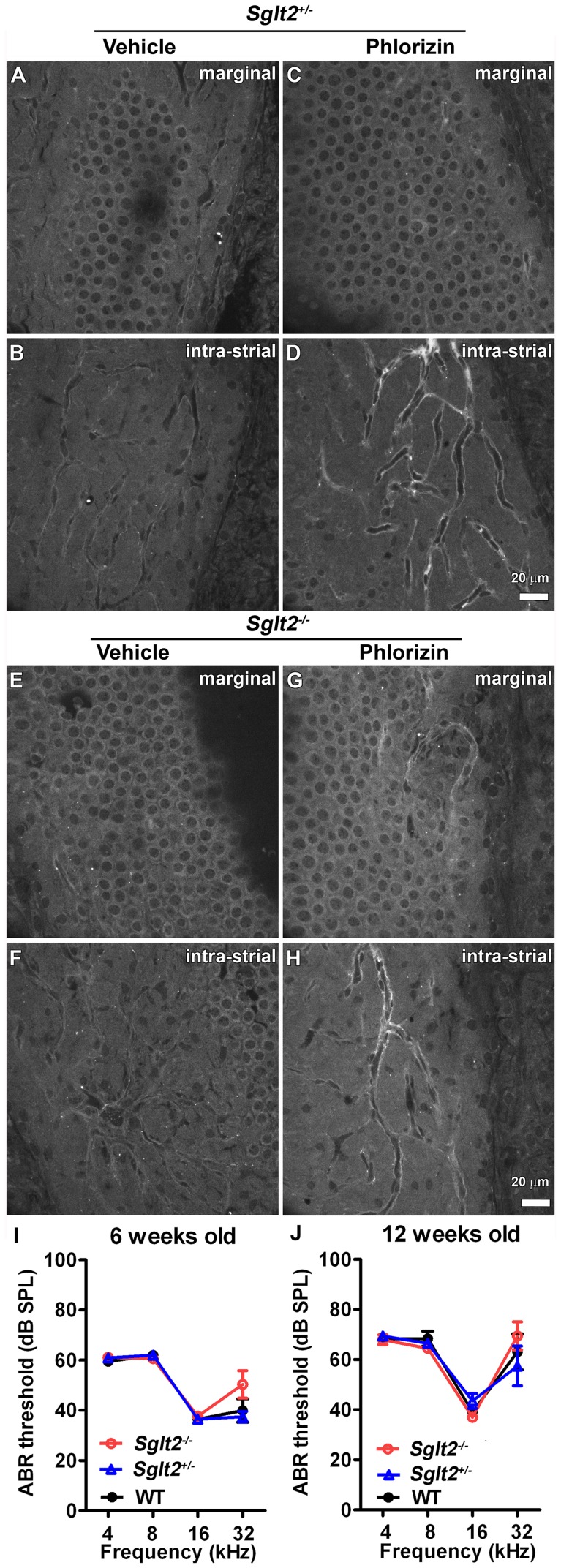
Loss of SGLT2 function had no effect on cochlear uptake of GTTR or auditory function. In the stria vascularis, GTTR was localized in marginal (A, E) and intermediate (B, F) cells of *Sglt2^+/−^* (A, B) and *Sglt2^−/−^* (E, F) mice. The nucleoplasm of marginal and intermediate cell nuclei displayed weak labeling. (C, D, G, H) Phlorizin had no effect on the uptake or distribution of GTTR fluorescence in the stria vascularis of *Sglt2^+/−^* or *Sglt2^−/−^* mice. Scale bar = 20 µm. (I, J) Wild-type, *Sglt2^+/−^* and *Sglt2^−/−^* mice, at 6 or 12 weeks of age, displayed no significant differences in ABR thresholds.

To test whether the low levels of SGLT2 immunofluorescence in the cochlea (Fig. 6) were required for cochlear uptake of GTTR, we examined whether phlorizin modulated the distribution of GTTR in murine cochleae of *Sglt2^+/−^* and *Sglt2^−/−^* mice. Mice were injected with GTTR 30 minutes after phlorizin or vehicle injection. In the stria vascularis, GTTR was characteristically localized in marginal and intermediate cells (Fig. 8) as previously described [35]. The nucleoplasm of marginal cell nuclei displayed negligible fluorescence (Fig. 8 A, C, E, G), as expected. There were no significant differences in the uptake or distribution of GTTR fluorescence between phlorizin- and vehicle-treated groups of *Sglt2^+/−^* or *Sglt2^−/−^* mice (Fig. 8 A–H). As observed in the kidney, SGLT2 was not required for cochlear uptake of GTTR in *Sglt2^−/−^* mice, and this uptake could not be inhibited by acute exposure to phlorizin in either *Sglt2^+/−^* or *Sglt2^−/−^* mice.

In wild-type mice, no statistically significant changes in auditory brainstem response (ABR) thresholds were observed after intraperitoneal (i.p.) injection with 800 mg/kg phlorizin or vehicle [DMSO; Fig. S4]. Furthermore, *Sglt2^−/−^* mice displayed no significant differences in ABR thresholds at 6 or 12 weeks of age compared to wild-type or *Sglt2^+/−^* (Fig. 8 I, J), and gender differences were minimal (Fig. S5). Thus, auditory function was not affected by phlorizin antagonism, or genomic loss of functional SGLT2.

### Toxicity studies

**Figure 9:**
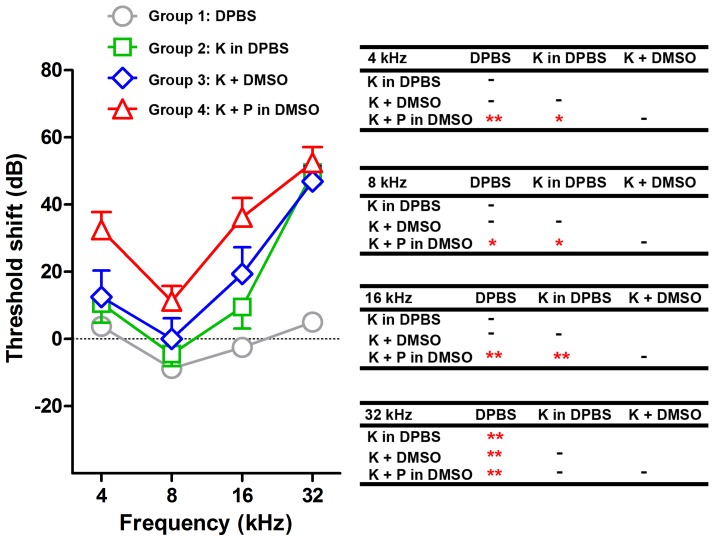
Phlorizin does not ameliorate kanamycin-induced ototoxicity. Three weeks after dosing, mice treated with kanamycin in DPBS or mice treated with kanamycin plus DMSO (vehicle for phlorizin) had significant ABR threshold shifts at 32 kHz only compared to mice treated with DPBS only (**p<0.01). The kanamycin plus phlorizin group had significantly different threshold shifts at 4, 8 and 16 kHz compared to the kanamycin in DPBS group; and significantly different threshold shifts at 4, 8, 16 and 32 kHz compared DPBS only group (*p<0.05; **p<0.01). However, no significant threshold shifts were observed between kanamycin plus phlorizin (in DMSO) and kanamycin plus DMSO groups.

Dosing wild-type mice with gentamicin *in vivo* causes systemic toxicity prior to induction of ototoxicity [48]. To test whether chronic phlorizin exposure ameliorates aminoglycoside cochleotoxicity as assessed by ABR threshold shifts, toxicity studies with another aminoglycoside, kanamycin, using a well-established protocol [48], in the presence or absence of phlorizin were conducted in wild-type mice. ABR threshold shifts were obtained before and after kanamycin dosing. In mice treated with just DPBS, insignificant threshold shifts were observed 3 weeks after dosing (Fig. 9; Fig. S6). In mice treated with kanamycin in DPBS, threshold shifts were observed at 32 kHz that were statistically significant compared with the DPBS-only group (Fig. 9; Fig. S6). In mice treated with kanamycin plus DMSO (vehicle for phlorizin), threshold shifts were observed at 16 and 32 kHz compared with the DPBS-only group, however, these thresholds shifts were significantly different only at 32 kHz (Fig. 9; Fig. S6). Mice treated with kanamycin plus phlorizin had statistically significant threshold shifts at 4, 8, 16 and 32 kHz compared with the DPBS-only group (Fig. 9; Fig. S6). The kanamycin plus phlorizin group also had threshold shifts were significantly different at 4, 8 and 16 kHz compared to the kanamycin in DPBS group (Fig. 9; Fig. S6). No significant differences in threshold shifts were observed between the kanamycin plus phlorizin and kanamycin plus DMSO groups (Fig. 9; Fig. S6). Thus, phlorizin did not protect auditory function from kanamycin-induced ototoxicity, and unexpectedly exacerbated drug-induced hearing loss at lower frequencies.

## Discussion

Here we report evidence, for the first time, that the electrogenic sodium-glucose transporter SGLT2 contributes to the cellular uptake of aminoglycosides, particularly by proximal tubule cells that highly express SGLT2 [17], [18], [56]. *In vitro*, SGLT2-mediated uptake of 2-NBDG and GTTR was inhibited by phlorizin and D-glucose. Cellular uptake of GTTR was enhanced by heterologous expression of SGLT2 in KDT3 cells. Knock-down of SGLT2 expression by siRNA reduced gentamicin-induced cytotoxicity in KPT2 cells endogenously expressing SGLT2, further suggesting SGLT2 involvement in cellular uptake of gentamicin and subsequent cytotoxicity. *In vivo*, we observed SGLT2 immunoexpression at the apical brush border region of kidney proximal tubule cells, and phlorizin pre-treatment can acutely inhibit GTTR uptake by proximal tubules in *Sglt2^+/−^* mice. Loss of SGLT2 function increased serum levels of gentamicin and GTTR. However, loss of SGLT2 function by phlorizin or gene knockout did not affect auditory function or cochlear uptake of GTTR. Phlorizin treatment exacerbated drug-induced hearing loss at lower frequencies.

Aminoglycosides enter cells, including kidney proximal tubule cells, via an endocytosis pathway [13], [57]–[60]. However, aminoglycoside exposure also generates reactive oxygen species within seconds in euthermic cells at room temperature, precluding endocytosis [61], [62]. Aminoglycosides also enter cells via non-selective cation channels, including TRP channels and the TRP-like mechanoelectrical transduction channels of sensory hair cells in the inner ear [15], [35], [51], [53], [63]. Identifying the molecular mechanisms by which aminoglycosides can enter cells, and particularly kidney proximal tubule and cochlear cells, is crucial to develop effective strategies to protect these pharmacologically-sensitive cells during clinically-essential gentamicin pharmacotherapy.

In the kidney, glomerular filtrate has ∼150 mM Na^+^ compared to intracellular levels of 12 mM, driving the inward electrogenic activity of Na^+^-ligand symporters present on the lumenal (apical) membrane of proximal tubule cells [55]. Proximal tubule cells also express high levels of SGLT2, a Na^+^-ligand symporter that traffics glycosides like glucose, facilitating the renal resorption of 90% of lumenal glucose from glomerular filtrate in promixal tubules [18], [19]. The kidney proximal tubule is also a primary location of aminoglycoside-induced nephrotoxicity [24], [25], [64].

In the cochlea, loop diuretics enhance the cochlear uptake of aminoglycosides [65]. Loop diuretics inhibit the Na^+^K^+^Cl^−^ co-transporter (NKCC) [66], predominantly localized on the basolateral membrane of marginal cells [65], [66], increasing the intra-strial concentration of Na^+^ [65]. If SGLT2 is localized on the basolateral membrane of marginal cells, as implicated by the discrete, yet low level, of immunoexpression within the stria vascularis, SGLT2 may be appropriately located to traffic aminoglycosides into marginal cells, prior to clearance into endolymph, and uptake by hair cells as shown previously [36]. As noted above, SGLT2 has a large, hydrophilic, elastic vestibule, with an internal pore diameter of ∼3 nm, and an exit pore (into cytosol) of ∼1.5–2.5 nm [26], [27] that is sufficiently large to potentially allow permeation by gentamicin. SGLT2 is blocked with high affinity by non-hydrolyzable glycoside derivatives that are generally well-tolerated acutely [30], [33], [67]. Thus, SGLT2 appeared to be rationally-identified candidate aminoglycoside transporter, and this hypothesis drove our experiments.

Fluorescently-conjugated gentamicin, GTTR, has been used to characterize the endocytotic trafficking of aminoglycosides to their intracellular domains in the kidney [12], [13]. Although the relative molecular mass (g/mol) and minimum cross-sectional diameter (mcd) of GTTR is larger than that of untagged gentamicin (gentamicin, 440–470 g/mol, mcd, 0.81 nm, GTTR, ∼1100 g/mol, mcd, ∼1.47 nm), GTTR can also permeate non-selective cation channels, with a sufficiently large pore diameter, directly into cytoplasm [15], [35], [51]. Using GTTR, we have previously shown that cytoplasmic uptake of GTTR can occur rapidly at low temperatures, precluding endocytosis, and is regulated by cellular potential, pH, extracellular cations (Ca^2+^, Gd^3+^, La^3+^), and non-specific cation channel blockers such as Ruthenium Red (RR), and verified using immunocytochemistry [15]. These properties are indicative of molecular permeation of ion channels, as for another fluorescent dye, FM 1–43 [68].

Using previously-described murine kidney cell lines [14], we observed specific SGLT2 immunofluorescence in proximal (KPT2), but not distal tubule (KDT3) cell lines, and in proximal tubules, but not distal tubules, *in vivo*. D-glucose is a primary substrate for SGLT2, which can also traffic the fluorescent glucose analog 2-NBDG. We found that a 1-fold molar excess of D-glucose competitively decreased 2-NBDG uptake by KPT2 cells. A 40-fold molar excess of D-glucose can also significantly decrease GTTR uptake of KPT2 cells. Thus, GTTR appears to have a greater affinity for SGLT2 than D-glucose and 2-NBDG. Uptake of 2-NBDG by KPT2 cells was also strongly inhibited by phlorizin, demonstrating robust SGLT2 activity in these cells, although not as efficaciously as D-glucose. Phlorizin also significantly decreased KPT2 uptake of GTTR and gentamicin in a dose-dependent manner by both immunofluorescence and ELISA. The uptake of GTTR was significantly attenuated ∼20% by phlorizin or Na^+^-free buffer, suggesting that this proportion of total GTTR uptake by KPT2 cells was mediated by SGLT2. Heterologous expression of SGLT2 in distal tubule-derived KDT3 cells significantly enhanced GTTR uptake, and this enhanced uptake can be abolished by phlorizin. The residual uptake of GTTR in these cells after phlorizin treatment likely represents GTTR uptake via previously-identified gentamicin-permeant cation channels, as demonstrated previously [14], [15], [69]. Furthermore, siRNA knockdown of SGLT2 expression in KPT2 cells reduced cellular susceptibility to gentamicin-induced cytotoxicity. Phlorizin had no apparent effect on the bactericidal activity of gentamicin on *E. coli* by disk diffusion assay. These *in vitro* data suggested that phlorizin, or other SGLT2 antagonists, may decrease cellular uptake of GTTR *in vivo*, and protect cells against aminoglycoside-induced cytotoxicity *in vivo*.

*In vivo*, we used two antisera for SGLT2 to determine if SGLT2 was appropriately located to contribute to aminoglycoside trafficking in the kidney and cochlea. Both antisera provided specific localization for SGLT2 in renal proximal tubules, with weak, less defined immunoexpression for SGLT2 in the cochlear stria vascularis (Fig. 5), and negligible SGLT2 immunolabeling in the organ of Corti. Although the SGLT2 inhibitor – phlorizin – acutely decreased GTTR uptake in proximal tubules of *Sglt2^+/−^* mice (Fig. 7), phlorizin did not inhibit cochlear uptake of GTTR of *Sglt2^+/−^* and *Sglt2^−/−^* mice (Fig. 8). It is not known whether phlorizin crosses the blood-brain barrier or the blood-labyrinth barrier, which may have limited its efficacy of blocking cochlear SGLT2. We also speculate (Fig. S7) that phlorizin inhibition, if any, of low cochlear levels of SGLT2-mediated GTTR trafficking would be compensated by phlorizin-induced elevation of GTTR serum levels (Fig. 7). Elevated serum levels of GTTR would increase trafficking into the cochlea via other residual cellular mechanisms of aminoglycoside uptake, e.g., endocytosis [12], [13] or ion channel permeation [14], [15]. However, genomic loss of SGLT2 function, unexpectedly, did not reduce GTTR uptake by proximal tubules compared to *Sglt2^+/−^* mice. Phlorizin did not alter proximal tubule uptake of GTTR in *Sglt2^−/−^* mice (Fig. 7), indicating that phlorizin had negligible effects on the cellular uptake of aminoglycosides in *Sglt2^−/−^* mice, acting specifically on SGLT2 in *Sglt2^+/−^* mice. Thus, renal GTTR uptake by *Sglt2^−/−^* mice likely occurs via compensatory mechanisms such as endocytosis or aminoglycoside-permeant cation channels, as discussed above. In addition, genomic loss of SGLT2 function and phlorizin-pretreatment in *Sglt2^+/−^* mice elevated serum levels of gentamicin or GTTR (compared to control-treated heterozygous *Sglt2^+/−^* mice; Fig. 7). This may be the result of a reduced volume of distribution for these compounds, and/or by a reduced glomerular filtration rate (GFR) [70], providing an alternate explanation for the reduced GTTR cytoplasmic and punctate fluorescence in proximal tubule cells. However, phlorizin did not alter serum levels of gentamicin or GTTR in *Sglt2^−/−^* mice (Fig. 7) due to the absence of a binding partner (*i.e.*, SGLT2) that facilitates aminoglycoside trafficking, further indicating the specificity of phlorizin for SGLT2.

There is no demonstrable nephrotoxicity or renal damage following kanamycin treatment (at 700–900 mg/kg per dose twice daily for 14 days) to induce ototoxicity in mice [48], as used here. To test for permanent changes in auditory performance, a recovery period of three weeks is optimal [48]. In mice, gentamicin at doses to induce ototoxicity causes systemic toxicity and mortality [48], [71]. Phlorizin or genomic loss of SGLT2 function did not affect auditory function, suggesting that SGLT2 activity is not required for cochlear function, and that glucose transport into the cochlea can be achieved by other transporters such as facilitated GLUTs [72], [73]. Whether GLUTs are aminoglycoside-permeant remains uncertain, as crystal structures have yet to be determined. Phlorizin, and genomic loss of SGLT2, did not reduce cochlear uptake of GTTR. Phlorizin did not protect auditory function from kanamycin-induced ototoxicity in wild-type mice. Crucially, neither phlorizin nor genomic loss of SGLT2 increased serum levels of aminoglycosides. Thus, we did not attempt to repeat the ototoxicity studies with *Sglt2^−/−^* mice.

In summary, SGLT2 increased cellular uptake of gentamicin and exacerbated gentamicin-induced cytotoxicity *in vitro*. Acute inhibition of SGLT2 function reduced gentamicin-induced cytotoxicity in kidney proximal tubule cells, *in vitro*, and may reduce the risk of gentamicin-induced nephrotoxicity in the kidney *in vivo*. Acute inhibition, but not chronic loss, of SGLT2 function reduced GTTR uptake by kidney cells *in vivo*. Loss of SGLT2 function increased serum levels of gentamicin and GTTR, but did not prevent cochlear loading, and can increase the risk of aminoglycoside-induced ototoxicity. These data suggest that clinical antagonism of SGLT2 function by phlorizin, and phlorizin derivatives like O-glycosides and C-glycosides, may be contra-indicated if patients are undergoing aminoglycoside therapy.

## Supporting Information

Figure S1 **SGLT2-mediated uptake of GTTR is attenuated by D-glucose.** Cells were treated with 5 µg/ml GTTR for 20 minutes at 37°C with increasing doses of D-glucose (molar ratios of 1∶0, 1∶40, 1∶2000 or 1∶40000 [GTTR/D-glucose])). Increasing doses of D-glucose reduced GTTR fluorescence in KPT2 cells. Scale bar = 20 µm.
(TIF)

Figure S2 **KDT3-SGLT2 cell line generation.** Parental KDT3, KDT3-SGLT2 and KDT3-pBabe cell lines have similar epitheloid morphology. Scale bar = 50 µm.
(TIF)

Figure S3 **Cell growth of control siRNA and SGLT2 siRNA transfected KPT2 cell.** MTT assay showed there was no difference for cell growth between control siRNA and SGLT2 siRNA transfected KPT2 cell at 1, 2 or 3 days after transfection.
(TIF)

Figure S4 **Phlorizin did not affect auditory function.** The ABR thresholds of wild-type mice 30 minutes after injection with 800 mg/kg phlorizin or vehicle (DMSO) control i.p., displayed no significant differences.
(TIF)

Figure S5 **Auditory function of** ***Sglt2^−/−^*** **mice.** Wild-type, *Sglt2^+/−^* or *Sglt2^−/−^* mice displayed no significant differences in ABR thresholds at 6 or 12 weeks of age. Male (blue) and female (red) displayed no significance differences.
(TIF)

Figure S6 **Auditory function by ABR before or 3 weeks after kanamycin treatment with or without phlorizin in wild-type mice.** In mice treated with kanamycin in DPBS, threshold shifts at 32 kHz were observed 1 day, post-treatment, 1.5 weeks post-treatment and 3 weeks post-treatment with kanamycin. In mice treated with kanamycin plus DMSO (vehicle for phlorizin), further threshold shifts were observed at 16 and 32 kHz at these 3 post-treatment time points. In mice treated with kanamycin plus phlorizin (in DMSO), threshold shifts were observed at 4, 16 and 32 kHz. Male (blue) and female (red) mice have little difference.
(TIF)

Figure S7 **Schematic representation of the effect of phlorizin on SGLT2-mediated GTTR uptake by the kidney or cochlea, as suggested by our data and interpretation.**
(TIF)

Table S1 **Phlorizin had no effect on bactericidal activity of gentamicin.** In *E. coli* disk diffusion assay, gentamicin (0.4 µg or 1 µg) alone induced a colony-free halo around the drug-impregnated disk, indicating baseline bactericidal effect. The colony-free diameter or halo thickness was not attenuated by increasing doses of phlorizin, indicating that phlorizin had no effect on the bactericidal activity of gentamicin.
(DOCX)
