# Supplementary figures and images for: Sodium-Glucose Transporter-2 (SGLT2; SLC5A2) Enhances Cellular Uptake of Aminoglycosides
Source: PLoS One. 2014 Sep 30;9(9):e108941. doi: 10.1371/journal.pone.0108941 (PMC4182564; doi:10.1371/journal.pone.0108941)

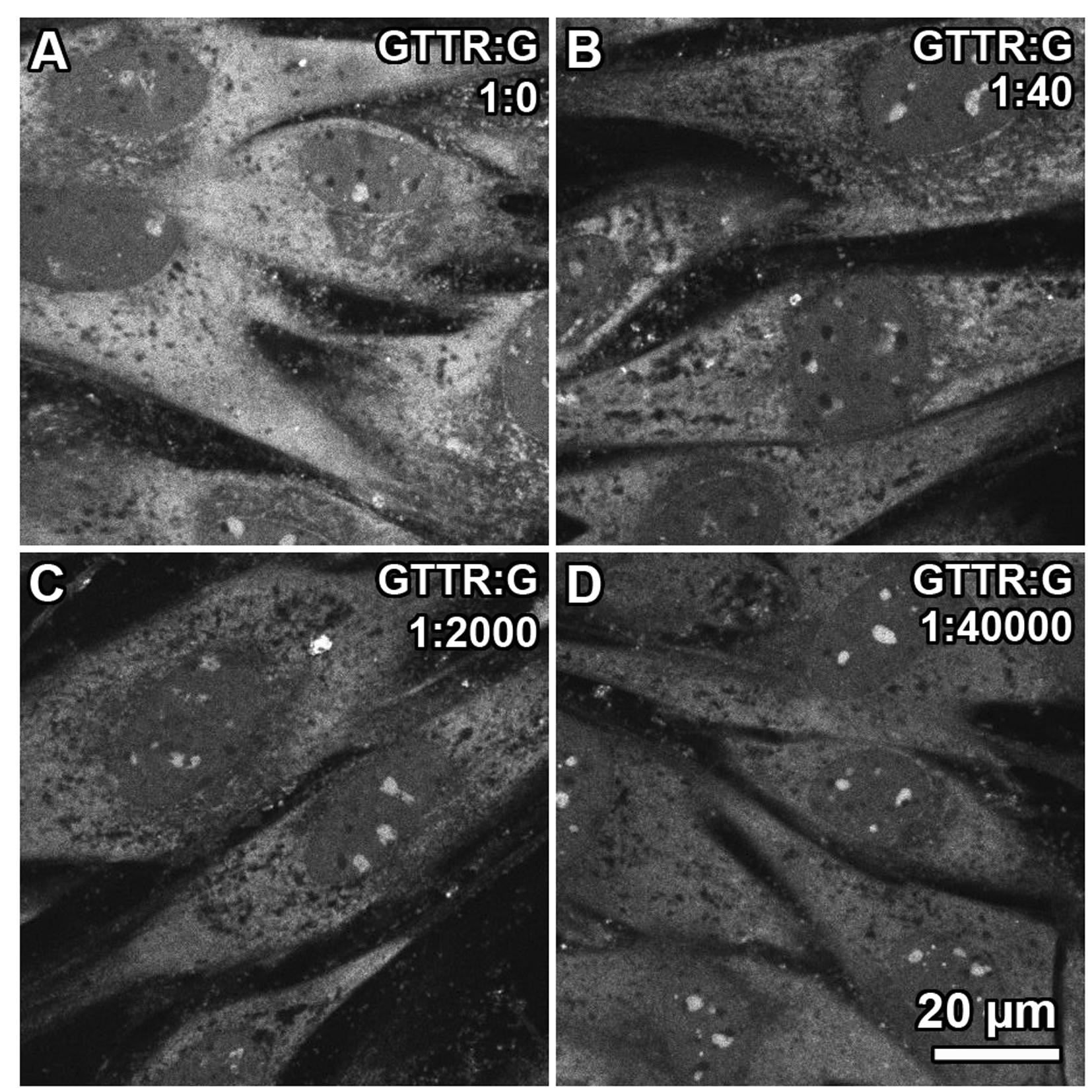

Supplement: Figure S1 — SGLT2-mediated uptake of GTTR is attenuated by D-glucose. Cells were treated with 5 µg/ml GTTR for 20 minutes at 37°C with increasing doses of D-glucose (molar ratios of 1∶0, 1∶40, 1∶2000 or 1∶40000 [GTTR/D-glucose])). Increasing doses of D-glucose reduced GTTR fluorescence in KPT2 cells. Scale bar = 20 µm. (TIF) [file pone.0108941.s001.tif]

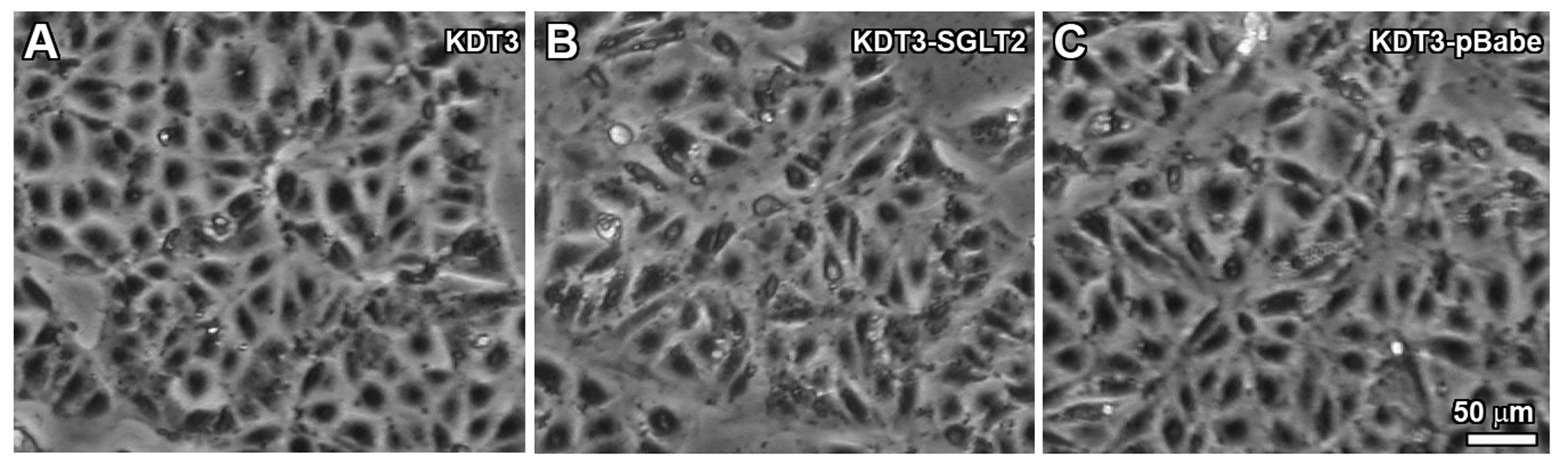

Supplement: Figure S2 — KDT3-SGLT2 cell line generation. Parental KDT3, KDT3-SGLT2 and KDT3-pBabe cell lines have similar epitheloid morphology. Scale bar = 50 µm. (TIF) [file pone.0108941.s002.tif]

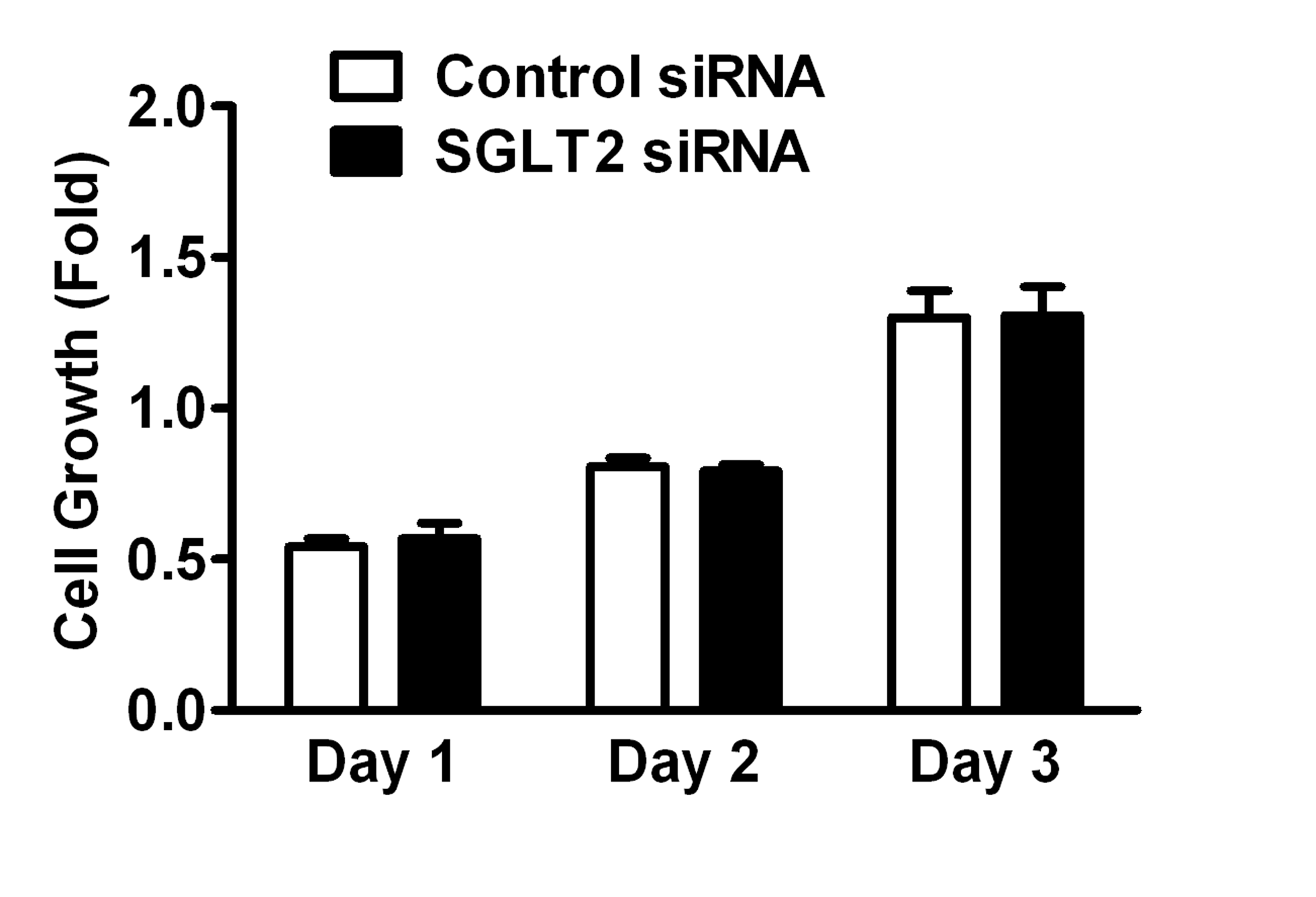

Supplement: Figure S3 — Cell growth of control siRNA and SGLT2 siRNA transfected KPT2 cell. MTT assay showed there was no difference for cell growth between control siRNA and SGLT2 siRNA transfected KPT2 cell at 1, 2 or 3 days after transfection. (TIF) [file pone.0108941.s003.tif]

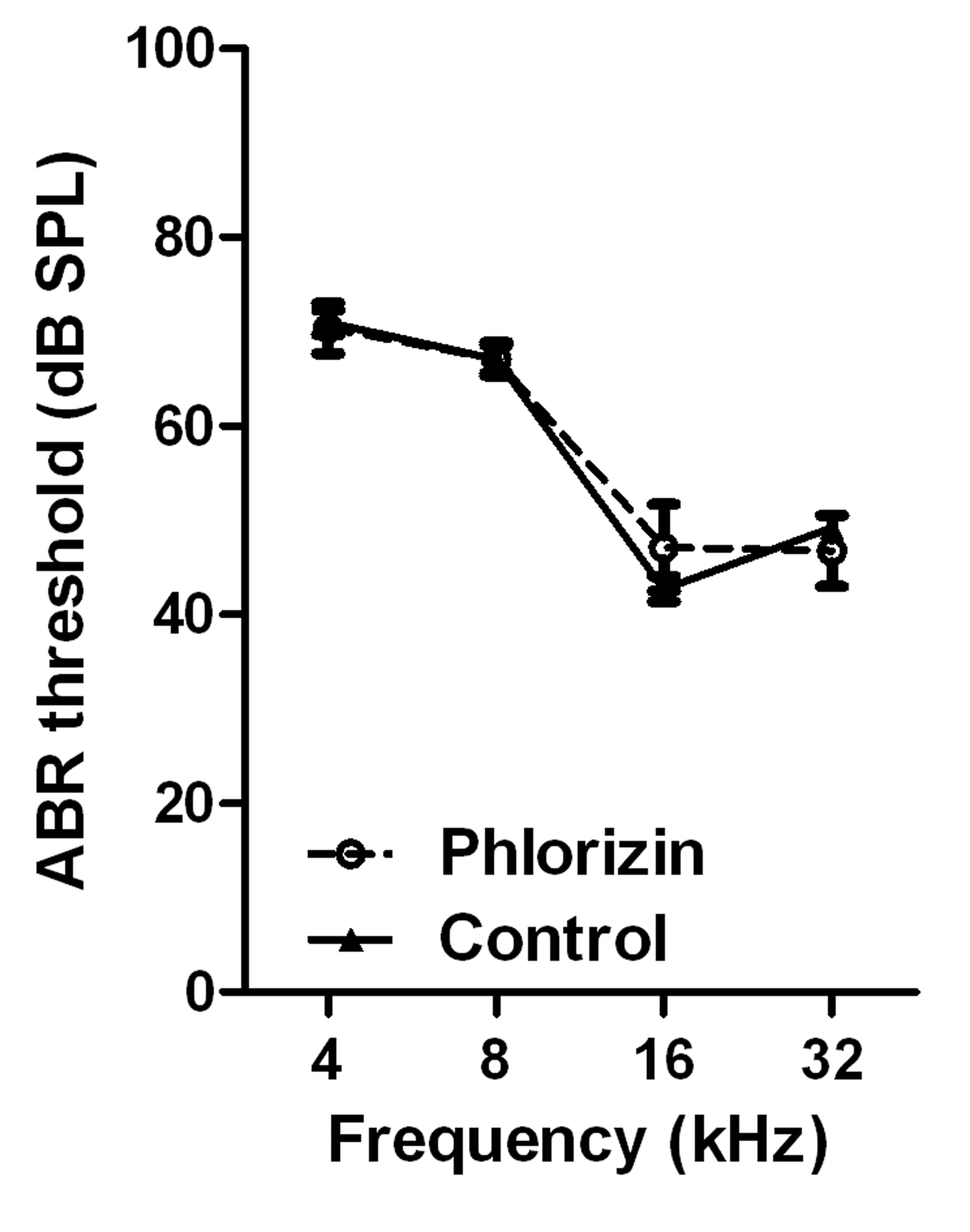

Supplement: Figure S4 — Phlorizin did not affect auditory function. The ABR thresholds of wild-type mice 30 minutes after injection with 800 mg/kg phlorizin or vehicle (DMSO) control i.p., displayed no significant differences. (TIF) [file pone.0108941.s004.tif]

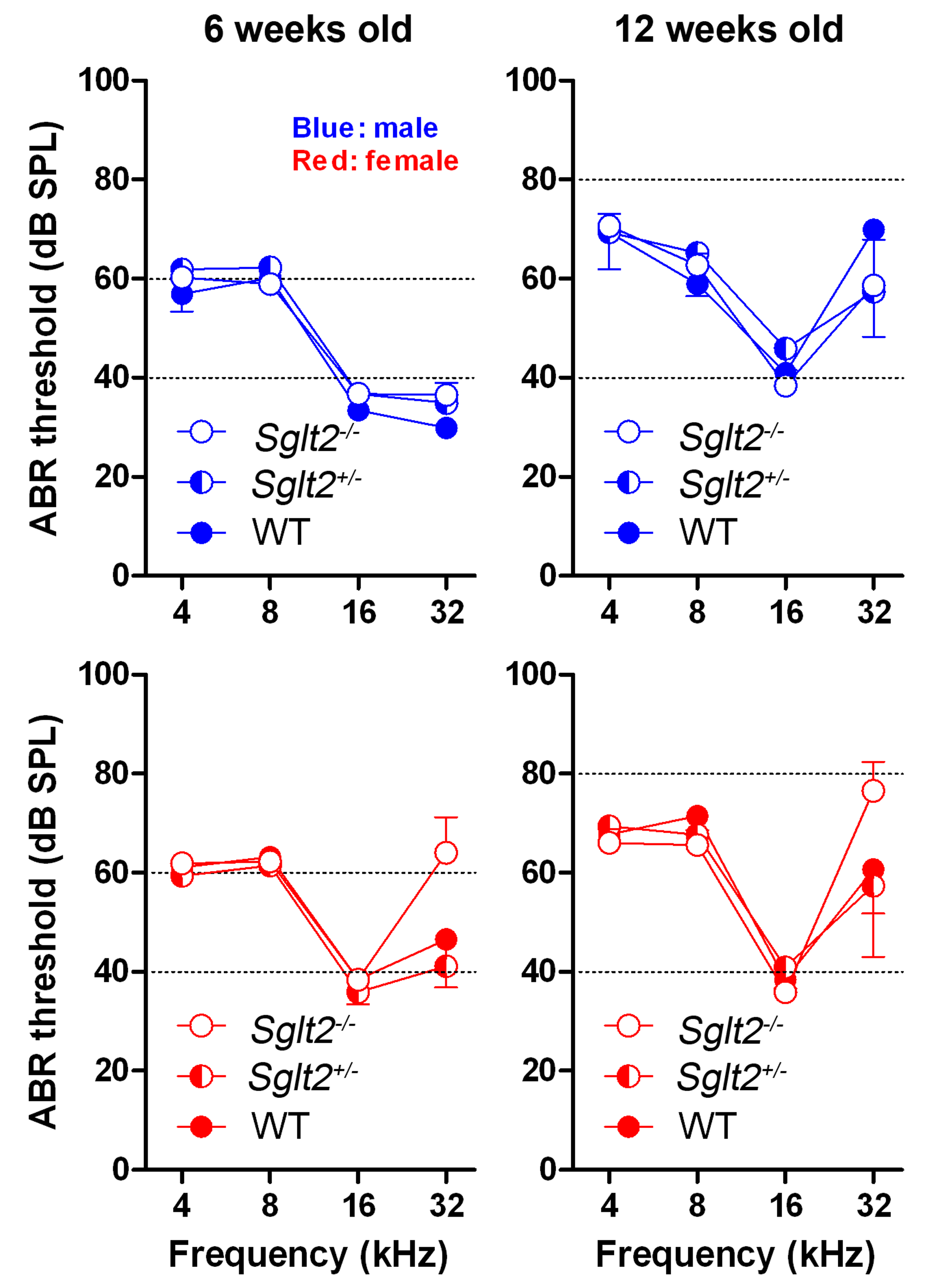

Supplement: Figure S5 — Auditory function of Sglt2−/− mice. Wild-type, Sglt2+/− or Sglt2−/− mice displayed no significant differences in ABR thresholds at 6 or 12 weeks of age. Male (blue) and female (red) displayed no significance differences. (TIF) [file pone.0108941.s005.tif]

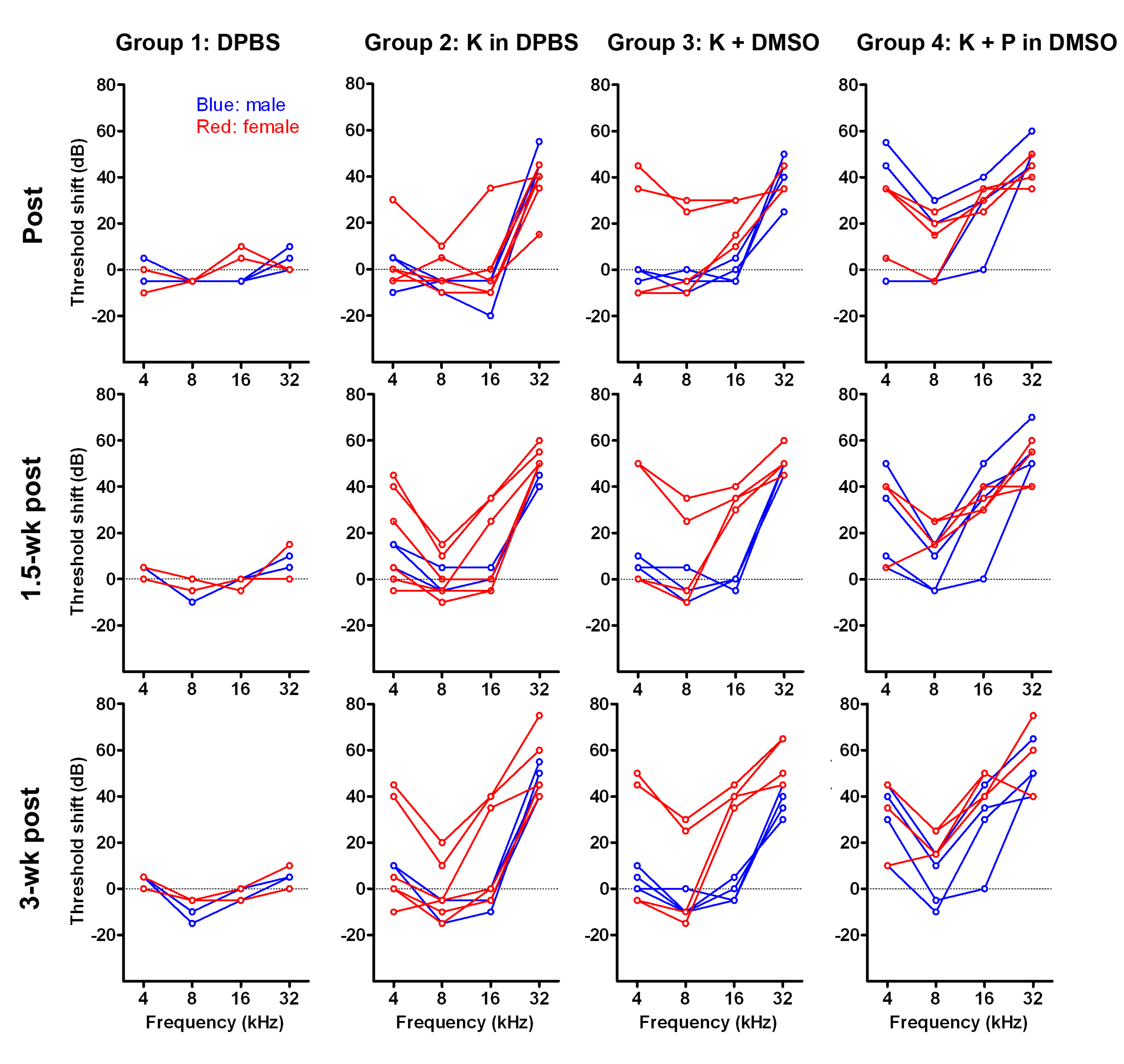

Supplement: Figure S6 — Auditory function by ABR before or 3 weeks after kanamycin treatment with or without phlorizin in wild-type mice. In mice treated with kanamycin in DPBS, threshold shifts at 32 kHz were observed 1 day, post-treatment, 1.5 weeks post-treatment and 3 weeks post-treatment with kanamycin. In mice treated with kanamycin plus DMSO (vehicle for phlorizin), further threshold shifts were observed at 16 and 32 kHz at these 3 post-treatment time points. In mice treated with kanamycin plus phlorizin (in DMSO), threshold shifts were observed at 4, 16 and 32 kHz. Male (blue) and female (red) mice have little difference. (TIF) [file pone.0108941.s006.tif]

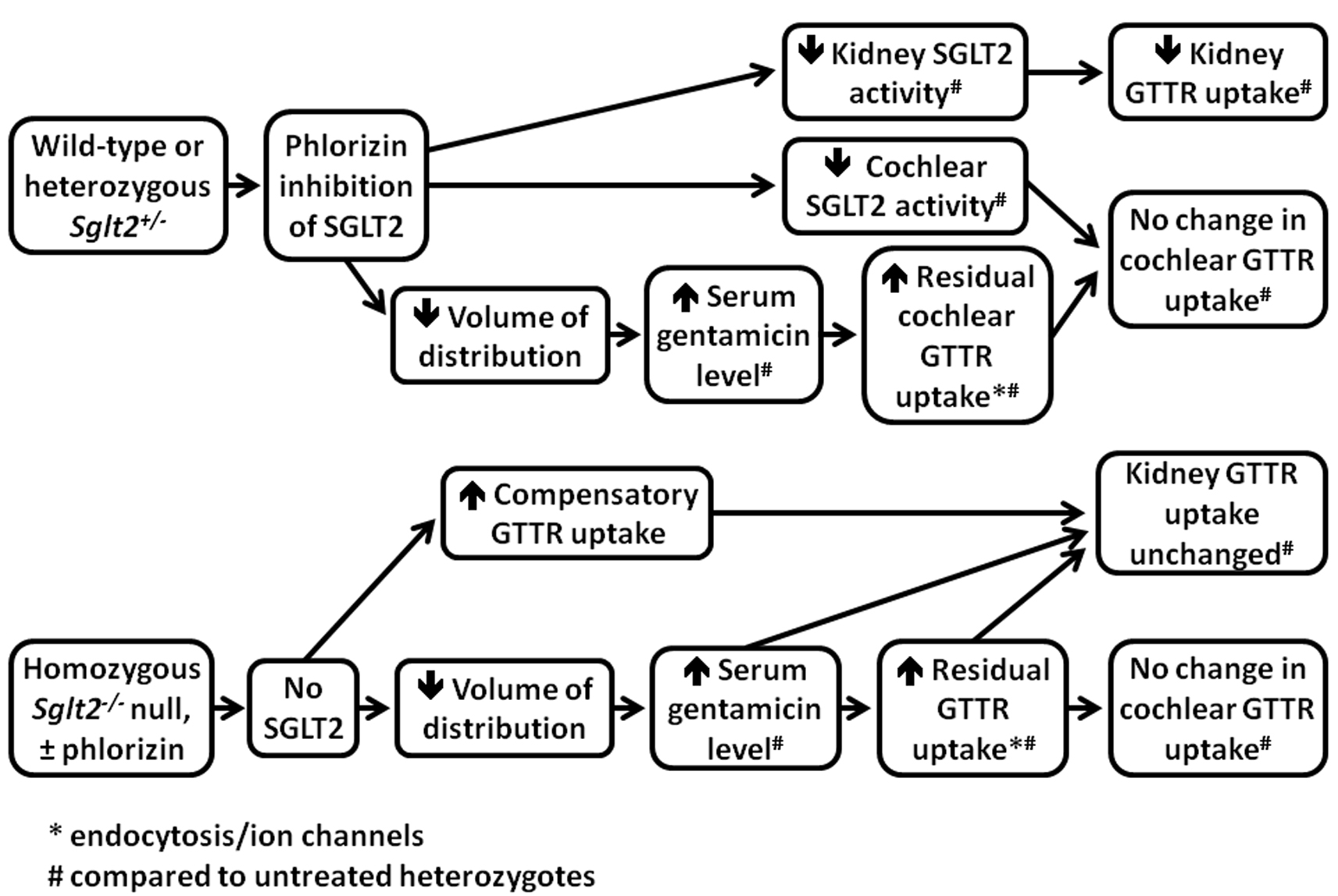

Supplement: Figure S7 — Schematic representation of the effect of phlorizin on SGLT2-mediated GTTR uptake by the kidney or cochlea, as suggested by our data and interpretation. (TIF) [file pone.0108941.s007.tif]
