# Supplementary material for: Sodium-Glucose Transporter-2 (SGLT2; SLC5A2) Enhances Cellular Uptake of Aminoglycosides
Source: PLoS One. 2014 Sep 30;9(9):e108941. doi: 10.1371/journal.pone.0108941 (PMC4182564; doi:10.1371/journal.pone.0108941)
Supplement: Table S1 — Phlorizin had no effect on bactericidal activity of gentamicin. In E. coli disk diffusion assay, gentamicin (0.4 µg or 1 µg) alone induced a colony-free halo around the drug-impregnated disk, indicating baseline bactericidal effect. The colony-free diameter or halo thickness was not attenuated by increasing doses of phlorizin, indicating that phlorizin had no effect on the bactericidal activity of gentamicin. (DOCX) [file pone.0108941.s008.docx]

**Table S1:** **Phlorizin has no effect on bactericidal activity of gentamicin**

| **GT (*µ*g)** | **Phlorizin (mg)** | **Diameter (mm)** | **Halo thickness (mm)** |
| --- | --- | --- | --- |
| **0.4** | **0** | **17.5** | **5.25** |
| **0.4** | **2** | **16.5** | **4.75** |
| **0.4** | **4** | **16** | **4.5** |
| **0.4** | **8** | **17** | **5** |
| **0.4** | **16** | **17** | **5** |
|  |  |  |  |
| **1** | **0** | **20.5** | **6.75** |
| **1** | **2** | **22** | **7.5** |
| **1** | **4** | **21** | **7** |
| **1** | **8** | **22** | **7.5** |
| **1** | **16** | **22** | **7.5** |
